# Tardigrades of Kristianstads Vattenrike Biosphere Reserve with description of four new species from Sweden

**DOI:** 10.1038/s41598-021-83627-w

**Published:** 2021-03-01

**Authors:** Edoardo Massa, Roberto Guidetti, Michele Cesari, Lorena Rebecchi, K. Ingemar Jönsson

**Affiliations:** 1 Department of Life Science, University of Modena and Reggio Emilia, via G. Campi 213/D, 41125 Modena, Italy; 2 Department of Environmental Science and Bioscience, Kristianstad University, 29188 Kristianstad, Sweden

**Keywords:** Zoology, Biodiversity

## Abstract

Kristianstads Vattenrike Biosphere Reserve [KVBR] is a UNESCO designated area of Sweden possessing high biological value. Although several studies on tardigrades inhabiting Sweden have been performed, the KVBR area has been neglected. The current study investigates the tardigrade fauna of five areas of the biosphere reserve and includes 34 samples of different substrates analysed quantitatively and qualitatively. In total, 33 species of tardigrades were found in the samples, including 22 new records for the Skåne region, 15 new records for Sweden, and four species new to science. *Mesobiotus emiliae* sp. nov., *Xerobiotus gretae* sp. nov., *Itaquascon magnussoni* sp. nov., and *Thulinius gustavi* sp. nov. were described with an integrative approach (when possible) using morphological characters (light, electron scanning, and confocal laser scanning microscopies) and molecular markers (ITS2, 18S, 28S, *cox1*). A new protocol to increase morphological data was developed recovering mounted specimens within old slides for SEM analysis. Emended diagnoses for the genus *Itaquascon* and the transfer of *Platicrista itaquasconoide* to the genus *Meplitumen* are proposed. This study enriches the knowledge of the tardigrade biodiversity both within the KVBR and in Sweden and contributes to the rapidly increasing number of tardigrade species reported worldwide. The 33 species identified in the KVBR area represents 28% of all water bear species found in Sweden so far. The restricted study areas and limited number of samples collected suggests that the KVBR is very rich of tardigrades.

## Introduction

In the last five years, more than 60 species of tardigrades new to science have been discovered and described^1^. Taking advantage of the integration of morphological and molecular techniques, taxonomists are now able to identify cryptic species, and to DNA barcode species, improving the accuracy of species description and identification. In spite of the ever-growing understanding of the diversity and phylogeny of the phylum Tardigrada, the faunistic data are still very scarce and highly under-studied. Only a small part of the publications reporting species new to science focuses on the composition of the community of tardigrades inhabiting a substrate or an area, e.g.^2–7^, even if the importance of combining taxonomic and faunistic studies have been suggested, e.g.^8–10^. Without extensive faunistic studies, e.g.^11–13^, information on the distribution (biogeography), auto- and syn-ecology, and adaptation of the species remain very limited.

In the past century, several studies on terrestrial and freshwater tardigrades from Sweden have been carried out^14–23^, reviewed by Guidetti et al.^24^. In total, 101 different species were reported from Sweden.

The main aim of the current study was to provide information about the tardigrade diversity of an unexplored area of Sweden, the Kristianstads Vattenrike Biosphere Reserve [KVBR] (Skåne County, Sweden). This biosphere reserve is a UNESCO designated area in the southernmost region of Sweden known for its wetlands, rivers, and lakes and for successful coadaptive management of its natural resources^25^. Within the Kristianstads Vattenrike, both residential areas and biotopes possessing high biological value of international and national importance such as wetlands, forest, and sandy arable land are included. In fact, this area of about 1050 km^2^ encompasses many sites of the “Natura 2000” network and nature reserves: it is one of the most biodiverse areas in Sweden, hosting 20% of the species considered threatened in the country^26^.

Despite the fact that KVBR is a hotspot of biodiversity, its tardigrade fauna is unknown.

We collected samples of different substrates from different sites within the KVBR, identifying morphospecies. These new records increased the checklist of the tardigrade Swedish fauna^24,27^ and underscored that the KVBR represents a hotspot for diversity. The identification of the specimens within the samples has been carried out with a morphological approach. We are aware that some of the species (e.g. those belonging to the *Ramazzottius, Milnesium, Paramacrobiotus, Macrobiotus*) identified in this study as morphospecies belong to complexes of cryptic species^28–31^. However, we consider appropriate to report the presence of morphospecies since the morphological adaptations of them give precious information on the diversity and structure of the communities, and on the ecology of the single species (or morphospecies) in relation to the substrates and sampled areas.

## Results

### Taxonomic and morphological results

Four species new to science (*Mesobiotus emiliae* sp. nov.*, Xerobiotus gretae* sp. nov.*, Itaquascon magnussoni* sp. nov.*,* and *Thulinius gustavi* sp. nov.) were found in the Kristianstad Vattenrike Biosphere Reserve, and their formal descriptions follow.

Morphometric data for the animals and the egg of these species are reported in the Tables 1 and 2 respectively (Supplementary Table S1 for the raw and the Thorpe’s normalized data). The morphometric data used in differential diagnoses of the species were not Thorpe’s normalized. After Thorpe’s normalization, only the lengths of very few characters in the new species resulted not isometric with respect to the length of the buccal tube (Supplementary Table S1). For these characters, the range (min–max) of *pt* indexes calculated on the Thorpe’s normalized data and the range (min–max) of *pt* indexes calculated on the non-normalized data did not change significantly (Supplementary Table S1).

**Table 1:** Summary of the morphometric data of the animals of the species new to science.

Number of measured specimens	*Mesobiotus emiliae* sp. nov 26		*Xerobiotus gretae* sp. nov 30		*Itaquascon magnussoni* sp. nov 16		*Thulinius gustavi* sp. nov 5	
Character	µm	*pt*	µm	*pt*	µm	*pt*	µm	*pt*
Body length	97–342	391–848	178–438	642–1068	136–509	657–1761	231–346	757–908
**Bucco-pharyngeal tube**								
Buccal tube length	24.76–40.31		27.67–43.60		19.84–31.12		26.10–38.84	
Pharyngeal tube length					18.34–34.44	91.58–113.80		
Bucco-pharyngeal tube length					41.01–64.75	190.66–212.79		
Buccal/pharyngeal tube length ratio (%)					88–109			
Stylet support insertion point	19.17–30.08	73.80–79.39	21.30–34.58	76.98–81.22	20.72–34.14	102.30–116.63	19.07–27.29	70.17–73.07
Buccal tube external width	3.40–7.64	13.73–19.64	3.61–6.38	11.96–16.08	2.86–5.72	11.67–19.09	3.66–5.20	12.53–14.02
Buccal tube internal width	2.60–6.28	10.50–16.14	2.35–4.41	7.35–11.12	2.14–4.71	8.90–15.67	2.61–3.83	9.08–10.71
Ventral lamina length	15.08–24.67	54.51–63.71	15.92–25.10	49.78–60.12				
**Placoid lengths**								
Macroplacoid 1	1.95–4.62	7.88–12.67	5.56–11.04	17.77–25.32			3.16–5.21	9.75–13.41
Macroplacoid 2	2.39–5.00	9.23–13.86	3.55–7.01	11.23–17.06			2.50–4.13	9.09–11.44
Macroplacoid 3	2.70–5.96	10.71–15.32					3.44–6.36	12.21–16.37
Microplacoid	1.93–4.45	7.29–12.34	1.41–3.68	4.14–8.44				
Macroplacoid row	8.72–17.88	35.22–45.96	9.89–18.36	34.77–43.33			9.88–16.97	35.53–44.36
Placoid row	11.48–22.57	46.37–61.11	12.79–22.82	43.73–53.35	9.91–17.21	44.82–63.54		
**Claw 1 heights**								
External base							5.71–8.62	21.06–24.08
External primary branch	5.46–7.99	19.41–22.88	6.11–8.77	17.66–22.70	7.62–12.28	30.96–43.31	9.25–12.39	30.28–35.44
External secondary branch	4.40–6.62	14.44–19.52	4.47–6.95	13.40–16.69	5.07–8.18	21.73–27.35	7.90–11.07	28.12–30.92
Internal base							4.90–7.22	13.99–19.24
Internal primary branch	5.22–7.57	17.10–23.33	5.80–8.27	17.12–21.65	6.21–10.93	28.45–35.87	9.12–11.95	29.66–34.94
Internal secondary branch	4.02–5.64	13.48–17.22	3.98–6.24	11.74–16.11	3.85–5.59	17.64–24.14	7.54–9.76	24.33–28.89
**Claw 2/3 heights**								
External base							6.56–8.07	21.28–25.13
External primary branch	5.56–8.97	20.40–25.39	6.58–10.84	19.50–26.06	8.61–16.42	38.14–55.34	10.79–14.70	32.51–41.34
External secondary branch	3.97–6.89	15.87–19.20	4.20–7.94	14.88–19.09	4.89–8.95	22.40–34.32	8.37–11.23	29.50–32.07
Internal base							5.33–6.77	14.89–22.34
Internal primary branch	4.82–8.67	19.06–23.77	6.04–9.90	18.01–24.07	6.67–12.05	30.55–42.95	8.86–11.60	29.72–33.95
Internal secondary branch	3.60–7.59	14.31–23.73	4.11–7.62	13.69–18.32	4.24–8.12	19.42–28.42	6.96–10.73	22.65–28.60
**Claw 4 heights**								
External base							6.43–9.79	23.11–27.38
External primary branch	6.07–9.65	20.70–25.95	5.56–8.87	16.29–21.72	6.11–13.05	27.99–45.04	10.70–14.50	36.42–41.00
External secondary branch	4.57–7.56	14.35–19.33	4.06–6.27	11.21–15.04	4.50–8.46	20.61–31.98	9.04–10.89	26.98–34.64
Internal base							8.18–10.11	25.40–31.34
Internal primary branch	6.10–9.88	23.45–29.07	6.29–10.10	17.48–24.13	8.34–17.22	38.20–56.51	12.71–16.73	41.55–48.70
Internal secondary branch	4.83–7.48	15.13–22.06	4.50–7.84	13.42–18.80	5.47–11.00	25.06–39.43	9.76–13.73	32.25–38.39

Within the table the number of specimens measured for each species (for the number of measurements for each character see Supplementary Table S1), the measures of the selected structures (characters; range in µm), and the relative *pt* indexes are displayed.

**Table 2:** Summary of the morphometric data of the eggs of two of the species new to science.

Character	*Mesobiotus emiliae* sp. nov				*Xerobiotus gretae* sp. nov			
	N	µm	MEAN	SD	N	µm	MEAN	SD
Egg bare diameter	3	46.9–64.6	58.6	10.1	2	86.2–89.1	87.7	2.1
Egg full diameter	3	62.4–76.5	70.8	7.5	2	97.5–102.6	100.1	3.7
Process height	9	7.9–10.6	9.0	0.8	6	4.7–5.7	5.2	0.4
Process base width	9	12.1–17.3	13.9	1.5	6	4.9–6.0	5.5	0.5
Process base/height ratio	9	144–164%	155%	8%	6	97–116%	107%	6%
Terminal disc width					6	2.5–3.6	3.0	0.4
Inter-process distance	9	0.5–1.6	1.1	0.4	6	1.0–2.7	1.6	0.6
Number of processes on the egg circumference	3	11.0–14.0	12.0	1.7	2	41.0–43.0	42.0	1.4

Within the table the measures of the selected structures (characters; range in µm), the number of measurements for each character, and the mean and the standard deviation calculated for each character measured.

**Mesobiotus emiliae sp. nov.**

ZooBank: lsid:zoobank.org:act:3DA2F1C0-BEC8-4D9A-B111-5D5F8F1FCCA0.

#### Type locality

Sånnarna, west of the nature reserve (Kristianstad, Skåne, Sweden). Sandy soil with grass (55.928931 N, 14.246299 E), collected on June 10th, 2014; sample SVC22 (C4342 in Bertolani’s Collection). The species was also found in two other localities (SVC34, 35) and it is probably present in further four localities in which only animals were found (SVC2, 8, 27, 30; Supplementary Table S2).

#### Type repositories

The holotype (SVC22 s11o), 34 paratypes, and an egg (SVC22 s1, s5, s7, s8, s11) are at Kristianstad University [HKR], 19 paratypes and an egg (SVC22 s4, s10) are in the collection of the Swedish Museum of Natural History [SMNH], and 3 paratypes and two eggs (C4342 s3, s9) are in the Bertolani’s Collection of University of Modena and Reggio Emilia [Unimore].

#### Description

**Figure 1:**
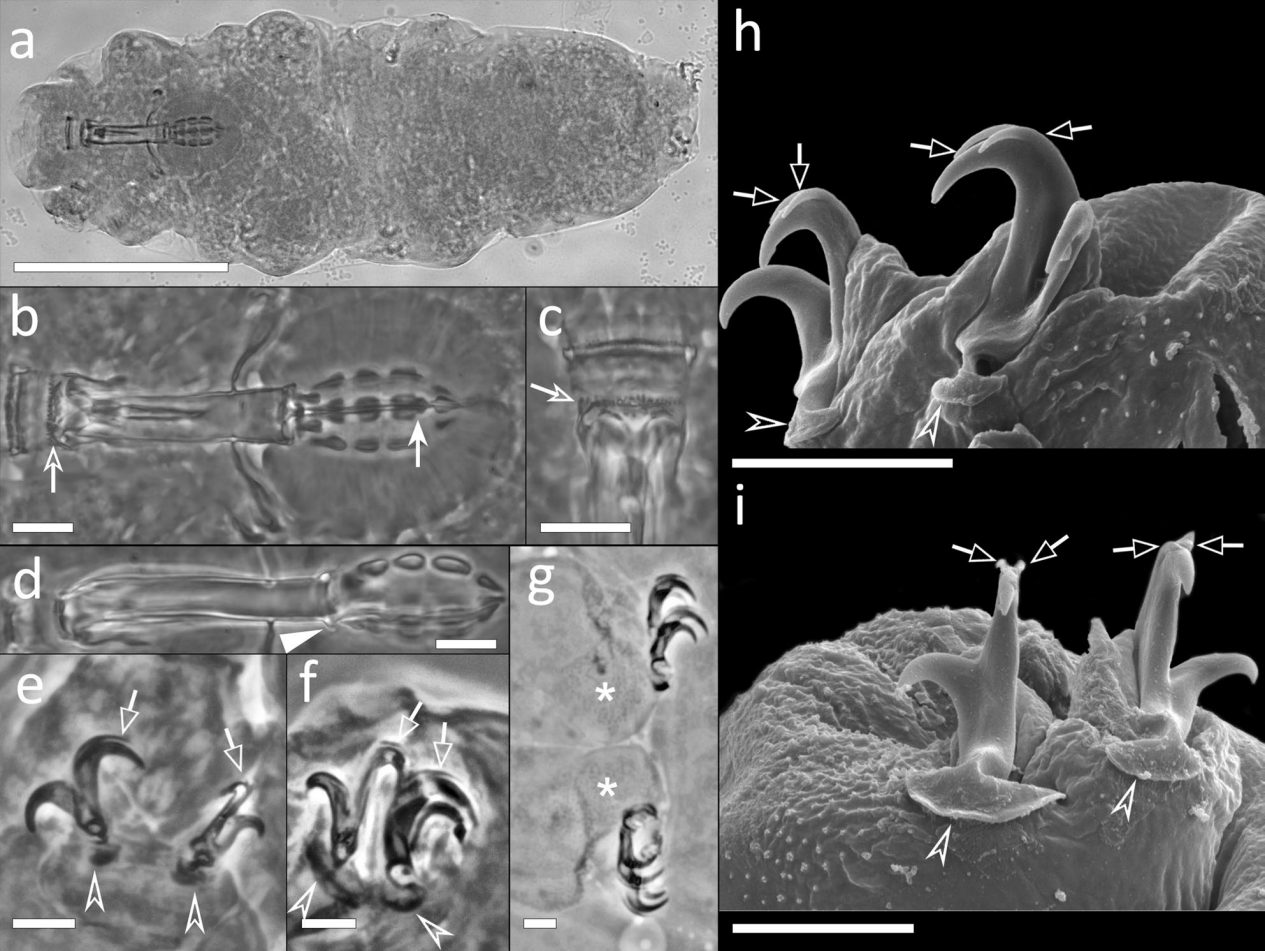
*Mesobiotus emiliae* sp. nov. (**a**) *In toto* (ventro-dorsal view) (**b**) Bucco-pharyngeal apparatus (dorso-ventral view from multiplanar images stack), (**c**) Buccal armature (ventral view), (**d**) Bucco-pharyngeal apparatus (lateral view) (**e**) Claw III (lateral view from multiplanar images stack), (**f**,**g**) Claw IV (lateral view from multiplanar images stack), (**h**) Claw III (fronto-lateral view), (**i**) Claw IV (frontal view). Empty indented arrows: crests of the buccal armature; indented white arrows: placoids constrictions; white arrowhead: cuticular ring of the buccal tube pharynx ending; empty arrows: accessory points of the main claw branch; empty indented arrowheads: lunules; asterisks: granulation on the legs IV. (**a**–**c**,**e**) Holotype. (**a–g**) LM, PhC; (**h**,**i**) SEM. Scale bars (**a**) 100 µm; (**b**–**d**) 10 µm; (**e**–**i**) 5 µm.

Body whitish, 96.8–342.0 µm in length (Fig. 1a). Eye-spots absent in mounted specimens. Cuticle smooth, with sparse granules on the posterior side of the legs IV (visible with Light Microscopy [LM]; Fig. 1g,i), and with granules covered with 1–5 dots on the external side of the legs I-III (Fig. 1h; visible with Scanning Electron Microscopy [SEM]).

Bucco-pharyngeal apparatus with antero-ventral mouth (Fig. 1b, d). Buccal ring with ten lamellae on its external margin. Buccal armature composed of: an anterior band of small teeth; a posterior line of conical teeth; three dorsal and three ventral transversal crests, the medio-ventral crest reduced to two or four mucrones in some smaller specimens (Fig. 1c), the latero-ventral crests shorter than the latero-dorsal ones. Short and straight stylet supports with distal flat enlargement, inserted at the 73.8–79.4% of the buccal tube. Typically-shaped stylet furca, with spherical condyles supported by short branches provided with small apophyses. Buccal tube ending with a thick cuticular ring within the pharynx (Fig. 1d). In the pharynx: pear-shaped pharyngeal apophyses; three grain-shaped (in lateral view) macroplacoids and an evident drop-shaped microplacoid. In frontal view, first macroplacoid triangular, second and third rectangular with rounded corners, and third with a deep distal constriction (Fig. 1b); length sequence 3 > 2 > 1.

Double-claws of *Mesobiotus* type (Fig. 1e–i) with evident accessory points on the main branch. All claws similar in shape, external claws slightly larger than internal. Claws increasing in size from the first to the fourth, claws of hind legs clearly the largest. Smooth lunules under all claws, larger under claws of the hind legs (the anterior lunules clearly larger with respect to the posterior).

**Figure 2:**
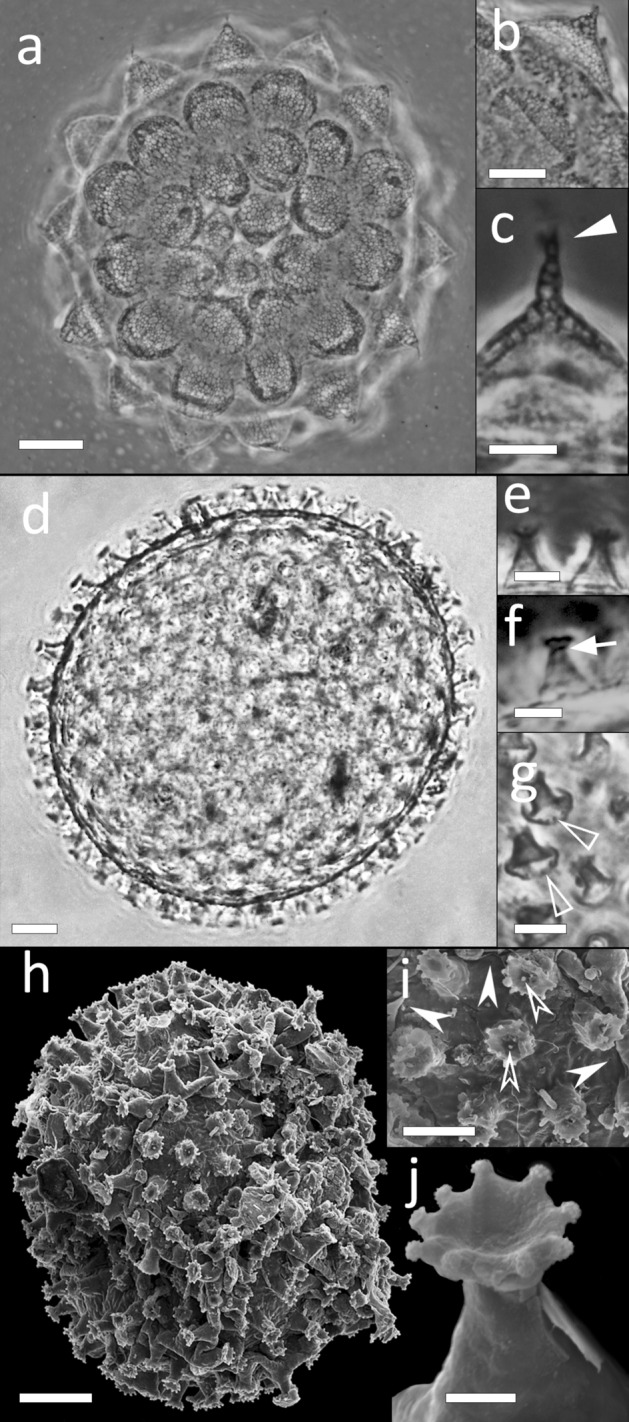
Eggs of *Mesobiotus emiliae* sp. nov. and *Xerobiotus gretae* sp. nov. (**a**–**c**) *M. emiliae* sp. nov. (**a**) *iIn toto*, (**b**) Processes surface detail, (**c**) Abnormal processes (lateral view). (**d–j**) *X. gretae* sp. nov. (**d**) *In toto*, (**e**,**f**) Processes (lateral view), (**g**) Processes (frontal view), (**h**) *In toto*, (**i**) Processes and surface detail, (**j**) Process (fronto-lateral view). Arrowhead: tuft of filaments on the tip of the egg process; arrow: septum dividing trunk and terminal disk of egg process; empty arrowhead: crown of dots at process base; indented arrowheads: pore on egg surface; empty indented arrowheads: indentation-like structures (provided with granular ornamentation) occurring in the upper surfaces of the disk. (**a–g**) LM, PhC; (**h**–**j**) SEM. Scale bars (**a**,**d**,**h**) 10 µm; (**b**,**c**,**e–g**,**i**) 5 µm; (**j**) 1 µm.

Spherical eggs free laid, ornamented with processes in shape of large and short cones or mammillated with tips of different lengths (generally short), sometimes terminating in a tuft of filaments (Fig. 2a–c). Process wall formed by two sides (an internal and an external), interspersed with trabecular structures forming irregular meshes (Fig. 2b), in shape of bubble-like structures in the longer process tips (Fig. 2c). Base of the processes with a crown of irregular and small thickenings: smaller thickenings in shape of large dots, the larger ones triangular-shaped (Fig. 2b). Filaments of the process tips mostly short, elongated in abnormal processes. Processes in numbers of 11–14 on the circumference. Egg surface between the processes smooth or sparsely dotted. Egg with embryos found.

#### Differential diagnosis

According to the taxonomic key of *Mesobiotus* species^32,33^*, M. emiliae* sp. nov. is different from any other described species of this genus. *Mesobiotus emiliae* sp. nov. belongs to the *harmsworthi* group of species, and within this group *Mesobiotus insuetus* (Pilato, Sabella & Lisi, 2014)^34^, *Mesobiotus lusitanicus* (Maucci & Durante Pasa, 1984)^35^*, Mesobiotus occultatus* Kaczmarek, Zawierucha, Buda, Stec, Gawlak, Michalczyk & Roszkowska, 2018^36^*, Mesobiotus patiens* (Pilato, Binda, Napolitano & Moncada, 2000)^37^*, Mesobiotus pseudoblocki* Roszkowska, Stec, Ciobanu & Kaczmarek, 2016^38^, and *Mesobiotus snaresensis* (Horning, Schuster & Grigarick, 1978)^39^ share with *Mesobiotus emiliae* sp. nov. the following characteristics: smooth cuticle; buccal armature with both anterior and posterior rows of teeth visible with LM, and without accessory teeth between posterior row and transversal crests; smooth lunules in the claws of the hind legs; eggs with processes in shape of cone or hemisphere with elongated tip or inverted funnel surrounded by a basal crown of dots or digitations, and with smooth or wrinkled surface between them.

*Mesobiotus emiliae* sp. nov. differs from:

*M. insuetus* by: the presence of granulation on legs IV, the shorter first macroplacoid (*pt* 15.4–16.8 in *M. insuetus*; *pt* 7.9–12.7 in *M. emiliae*), the shorter macroplacoids row (*pt* 46.2–48.9 in *M. insuetus*; *pt* 35.2–46.0 in *M. emiliae*), the different morphology of the hind claws (i.e. not with secondary branches diverging distally and forming a right angle with the primary branches as in *M. insuetus*), the shorter primary branch of all claws (e.g., *pt* of the primary branch of the claws II, 31.9–36.0 in *M. insuetus*; 20.4–25.4 in *M. emiliae*), and the processes of the egg with larger meshes on the surface (with LM, as small separated dots in *M. insuetus* and bubbles in contact to each other in *M. emiliae*);

*M. lusitanicus* by: the presence of granulation on legs IV, the length sequence of macroplacoids (3 > 1 > 2 in *M. lusitanicus*), the more evident microplacoid (*pt* 4.2 in *M. lusitanicus*; *pt* 7.3–12.3 in *M. emiliae*), and the shape of egg processes (i.e. not in shape of hemispheres terminating with a cap-like structure or with a fringed cones as in *M. lusitanicus*);

*M. occultatus* by: the absence of granulation on the legs I-III (with LM), the length sequence of macroplacoids (1 ≥ 3 > 2 in *M. occultatus*), the smaller eggs (full diameter 97.4–126.6 µm in *M. occultatus* and 62.4–76.5 µm in *M. emiliae*), the shape of the egg processes with a base/height ratio (74–106% in *M. occultatus* and 144–164% in *M. emiliae*), the distance between the egg processes (mean 2.6 µm, 0.6 SD in *M*. *occultatus* and mean 1.1 µm, 0.4 SD in *M. emiliae*);

*M. patiens* by: the absence of granulation on the legs I-III (with LM), the length sequence of macroplacoids (1 > 3 > 2 in *M. patiens*), the smaller eggs (full diameter 90.5–100.0 µm in *M. patiens* and 62.4–76.5 µm in *M. emiliae*), absence of slender tips in the egg processes;

*M. pseudoblocki* by: the presence of granulation on legs IV, the length sequence of macroplacoids (1 > 3 > 2 in *M. pseudoblocki*), the smaller anterior claw of the hind legs (*pt* 27.5–33.5 in the primary branch and 19.8–27.4 in the secondary branch in *M. pseudoblocki* and *pt* 27.5–33.5 in the primary branch and 19.8–27.4 in the secondary branch in *M. emiliae*), the closer processes on the egg surface (mean 2.8 µm, 0.6 SD in *M. pseudoblocki* and mean 1.1 µm, 0.4 SD in *M. emiliae*), the processes of the egg not in shape of sharpened narrow cones, the processes base/height ratio (47–70% in *M. pseudoblocki* and 144–164% in *M. emiliae*);

*M. snaresensis* by: the presence of granulation on hind legs, the more evident microplacoid (*pt* 4.2–7.3 in *M. snaresensis*; *pt* 7.3–12.3 in *M. emiliae*), the processes of the egg not terminating with a sharp or bifid tips, and the absence of pseudoareolation on the egg surface between the processes.

#### Molecular characterization

The analyses of the molecular markers were not possible due to the lack of alive specimens: the genomic material extracted from dead specimens gave no amplicons.

#### Etymology

We dedicate this species to Emilia Lonis, the beloved hundred-years-old grandmother of the coauthor Massa E., one of the last living workers that with their hand-on work for the reclamation of the “Piana di Terralba” (Sardinia, Italy; now site of the “Natura 2000” network) have contributed to the eradication of malaria in the island saving thousands of life.

**Xerobiotus gretae sp. nov.**

ZooBank: lsid:zoobank.org:act:E5265E82-86E9-4069-9B3B-B0F11D43DE79.

#### Type locality

Sånnarna, (Kristianstad, Skane, Sweden). Moss on ground (55.928056 N, 14.252694 E), collected on June 10th, 2014. Sample SVC15 (C4341 in the Bertolani’s Collection). An animal of this species was also found within a *Saxifraga* sp. (SVC19; Supplementary Table S2).

#### Type repositories

The holotype (SVC15 s2m), 51 paratypes, and an egg (SVC15 s2, s3) are at HKR, 50 paratypes (SVC15 s5) are in the collection of the SMNH, and 29 paratypes and an egg (C4341 s1, s4) are in the Bertolani’s Collection of Unimore. Five paratypes recovered from the old slide C4341 s1 (for the extraction and mounting protocol, see: Methods section) together with four paratypes and an egg freshly extracted were mounted on stubs for SEM observation.

#### Description

**Figure 3:**
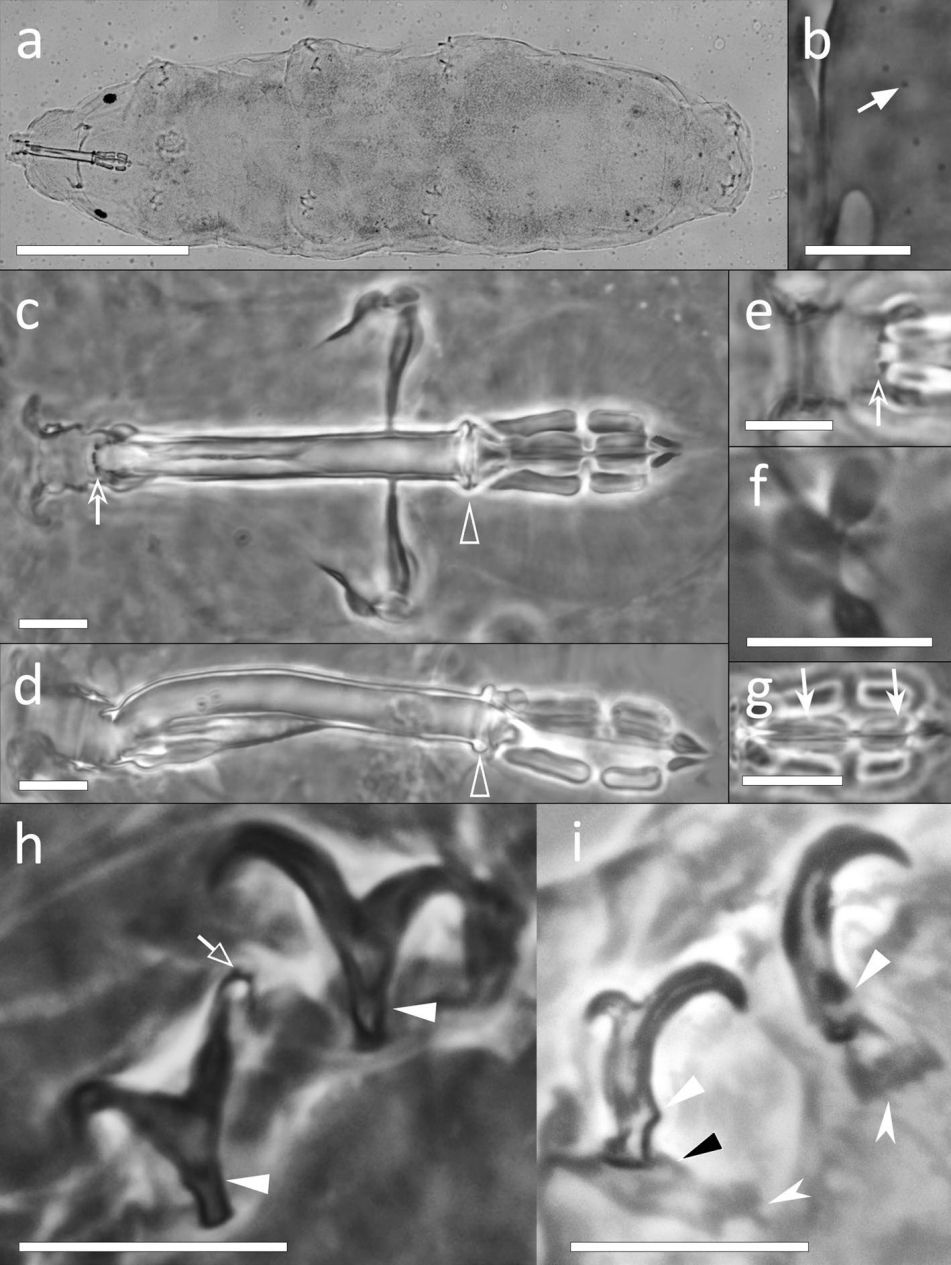
*Xerobiotus gretae* sp. nov. (**a**) *In toto* (ventro-dorsal view), (**b**) Cuticular ornamentation (dorsal view), (**c**) Bucco-pharyngeal apparatus (dorso-ventral view from multiplanar images stack), (**d**) Bucco-pharyngeal apparatus (lateral view from multiplanar images stack), (**e**) Buccal armature (ventral view), (**f**) Stylet furca (frontal view), (**g**) Macroplacoids (frontal view from multiplanar images stack), (**h**) Claw III (frontal view), (**i**) Claw VI (lateral view). Arrow: cuticular pores; empty indented arrows: crests on the buccal armature; empty arrowheads: cuticular ring of the buccal tube pharynx ending; indented white arrows: placoid constrictions; empty arrow: accessory points of the main claw branch; arrowheads: basal rounded cuticular thickening of the claws; black arrowhead: lunules; indented white arrowheads: cuticular bars. (**a,c**) Holotype; (**a**–**i**) LM, PhC. Scale bars (**a**) 100 µm; (**b**–h) 10 µm.

**Figure 4:**
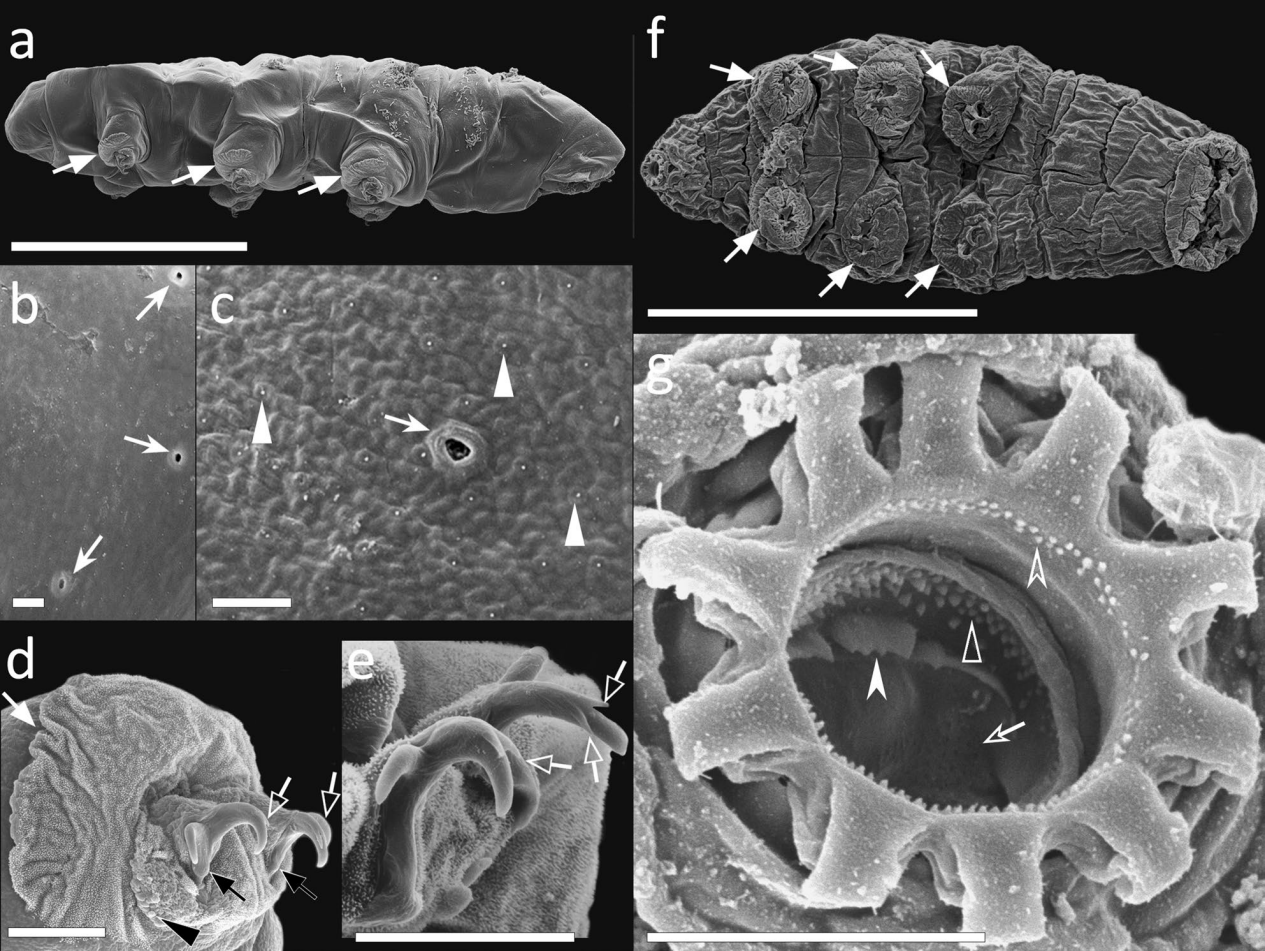
*Xerobiotus gretae* sp. nov. (**a**–**e**) Specimens mounted on stub from alive tardigrades. (**f**–**g**) Specimens mounted on stub from old permanent slide. (**a**) *In toto* (lateral view), (**b**,**c**) Cuticle (dorsal view), (**d**) Claw I (fronto-lateral view), (**e**) Claw IV (lateral view), (**f**) *In toto* (ventral view), (**g**) Buccal opening. White arrows: garter-like structure covered with microdigitations; indented white arrows: pores; arrowheads: very small single dots on the cuticle; empty arrows: accessory points; black arrows: basal rounded cuticular thickening of the claws; black arrowhead: granules on the garter-like structure; empty indented arrowhead: anterior band of small teeth at the proximal end of the peribuccal lamellae; empty arrowhead: posterior line of small teeth; indented white arrowhead: dorsal transversal crests; empty indented arrow: cribrose area in buccal tube. SEM. Scale bars (**a**,**f**) 100 µm; (**b**,**c**) 1 µm; (**d**,**e**,**g**) 5 µm.

Body whitish or pale green, 177.5–438.3 µm in length (Figs. 3a, 4a,f). Orange eye-spots present in mounted specimens. Very small scattered pores (about 0.5 µm in diameter) in the dorso-lateral cuticle (Figs. 3b, 4b,c). Very small single granules, distributed almost regularly, present on the entire cuticle (only visible with SEM; Fig. 4c). Legs of the first pair smaller than those of the second and third pairs. The area of the leg cuticle surrounding the claws with a swelling (forming a garter-like structure; Fig. 4a, d–f). These swellings appearing covered with microdigitations and few minute scattered granules (with SEM; Fig. 4d).

Bucco-pharyngeal apparatus with antero-ventral mouth (Fig. 3c,d). Buccal ring with ten peribuccal lamellae (Fig. 4g). Buccal tube of *Macrobiotus* type*,* curved in the first half, and ending with a thick cuticular ring within the pharynx (Fig. 3c,d). Ventral lamina with an antero-ventral thickening (Fig. 3d). Buccal armature (Figs. 3c,e, 4g) composed of: an anterior band of small teeth at the base of the peribuccal lamellae (with SEM); a thin posterior band of small teeth not always visible with LM, but clearly visible with SEM; three dorsal and three ventral transversal crests, medio-ventral crest appearing split in two or three mucrones in some specimens (with LM). Lateral cribrose areas posterior to the transversal crests visible with SEM (Fig. 4g). Stylet support, inserted at 77.0–81.2% of buccal tube, in shape of an elongated sigma with a distal flat enlargement (Fig. 3c). Typically-shaped stylet furcae, with oval condyles supported by short branches provided with rounded apophyses (Fig. 3f). In the pharynx: large and triangular pharyngeal apophyses overlapping the first macroplacoid; two rod-shaped macroplacoids (in lateral view; Fig. 3d), and evident drop-shaped microplacoid. In frontal view (Fig. 3g), the first macroplacoid in shape of a drop with a medial slight constriction longer than the second, the second rectangular with rounded corners and with a small terminal slight constriction.

Double-claws I-III different from claws IV (Figs. 3h,i, 4d,e): claws I–III of *Xerobiotus* type (without lunules), claws IV with a longer common tract, small and short claw branches, and pale lunules (more sclerified proximally than distally) sometimes visible (Fig. 3i). Internal and external claws of the same leg similar in shape, external (or posterior, in claw IV) claw slightly larger than the internal (or anterior, in claw IV). Proximal portion of the basal part of all claws with a small enlargement, larger in claw IV (Figs. 3h,i, 4d). Claws increasing in length from the first to the third pair. Primary branch of all claws with short accessory points (larger in claws IV). Cuticular bars under the base of the claw IV thick and with ragged margin (Fig. 3i): cuticular bar under the posterior claw wider and stretched toward the anterior claw, cuticular bar under anterior claw developed toward the front of the body (Fig. 3i).

Spherical eggs laid free (Fig. 2d,h), ornamented with processes in shape of inverted goblets with straight or concave cross section (according to Kaczmarek et al.^35^; Fig. 2e–g,i,j). Processes base surrounded by a crown of dots (Fig. 2g); terminal disc slightly concave and divided by a septum from the trunk (Fig. 2e,f). The edge of the terminal disks indented, the indentations appearing like tapered tip (with LM; Fig. 2g) and like elongated processes ornamented with granules (with SEM; Fig. 2i); in several processes indentation-like structures (provided with granular ornamentation) occurring also in the upper surfaces of the disk (Fig. 2j). Wrinkled egg surface between the processes (Fig. 2g, i) and scattered with dot-like pores (with SEM; Fig. 2i). Egg with an embryo found.

#### Differential diagnosis

*Xerobiotus gretae* sp. nov. differs from all other *Xerobiotus* species by having pores on the cuticle visible with LM, an enlargement in the basal part of the claws, and cuticular bars under the claws IV.

Moreover, *Xerobiotus gretae* sp. nov. differs from:

*Xerobiotus xerophilus* (Dastych, 1978)^41,42^ by: the presence of a posterior band of teeth and the dorsal transversal crests not fused in the buccal armature, the shape of the egg processes (flattened hemispherical processes in *X. xerophilus*), and the egg surface lacking reticulation;

*Xerobiotus euxinus* Pilato, Kiosya, Lisi, Inshina & Biserov, 2011^43^ by: the dorsal transversal crests not fused in the buccal armature, and the presence of cuticular bars under the claws of the hind legs;

*Xerobiotus pseudohufelandi* (Iharos, 1966)^44^ by: the presence of a posterior band of teeth, shorter common tract in the claws I-III (*pt* 11.07–11.99 in *X. pseudohufelandi*; *pt* 9.19–9.91 in *X. gretae*), and the egg surface lacking reticulation.

#### Molecular characterization

It was not possible to extract genetic material from the specimens recollected from the permanent slides (C4341 A–E). The analyses of the molecular markers amplified from four specimens (C4341 G–L; GenBank accession number: MW581665-8, *cox*1; MW588431-3, ITS2; MW588438-41, 28S; MW588434-7, 18S; Supplementary Table S5) revealed single haplotypes for ITS2, 18S, and 28S genes, and three haplotypes for *cox1* gene (highest p-distance = 0.4%; Supplementary Table S3).

*Xerobiotus gretae* sp. nov., in comparison to the more similar GenBank sequences which belong to *Xerobiotus* sp. collected in South Africa (Cape of Good Hope, Western Cape)^45^, differs for p-distances of 1.6–2.6% for *cox1* (796 bp), 1.0% for ITS2 (452 bp), and 0.0% for 18S (870 bp). *Xerobiotus pseudohufelandi,* collected in Italy (Monte Calvario)^46^, differs from *X. gretae* sp. nov. for p-distance of 16.9–17.8% for *cox1*, and 0.0–0.1% for 18S, *Xerobiotus* sp., collected in Poland (Błedowska Desert)^45^, differs from *X. gretae* sp. nov. for p-distances of 17.8–18.1% for *cox1*, 5.1% for ITS2, and 0.1–0.2% for 18S (Supplementary Table S3).

#### Etymology

We dedicate this species to the climate activist Greta Thunberg, for her brave and insightful efforts to open the eyes of the world leaders about the need for action against climate change. The achievements of Greta Thunberg give us hope that the challenges of changing the unsustainable path of human societies may still be possible, just like the tiny tardigrades are able to overcome seemingly impossible environmental challenges. But we have to act now!

**Itaquascon magnussoni sp. nov.**

ZooBank: lsid:zoobank.org:act:254843BC-60F7-4B8E-A94D-8783214F3399.

#### Type locality

Näsby Fält (Kristianstad, Skåne, Sweden), along a trail to Araslövssjön Lake. Moss on bark of *Alnus* sp. (56.059328 N, 14.136678 E)*,* 2 m up on the tree, collected on June 10th, 2014. Sample SVC32 (C4344 in the Bertolani’s Collection). The species was also found in two other localities (SVC3, 27; Supplementary Table S2).

#### Type repositories

The holotype (SVC32 s4c) and 13 paratypes are at HKR, four paratypes (SVC32 s8) are in the collection of the SMNH, and eight paratypes (C4344 s12) in the Bertolani’s Collection of Unimore.

#### Description

**Figure 5:**
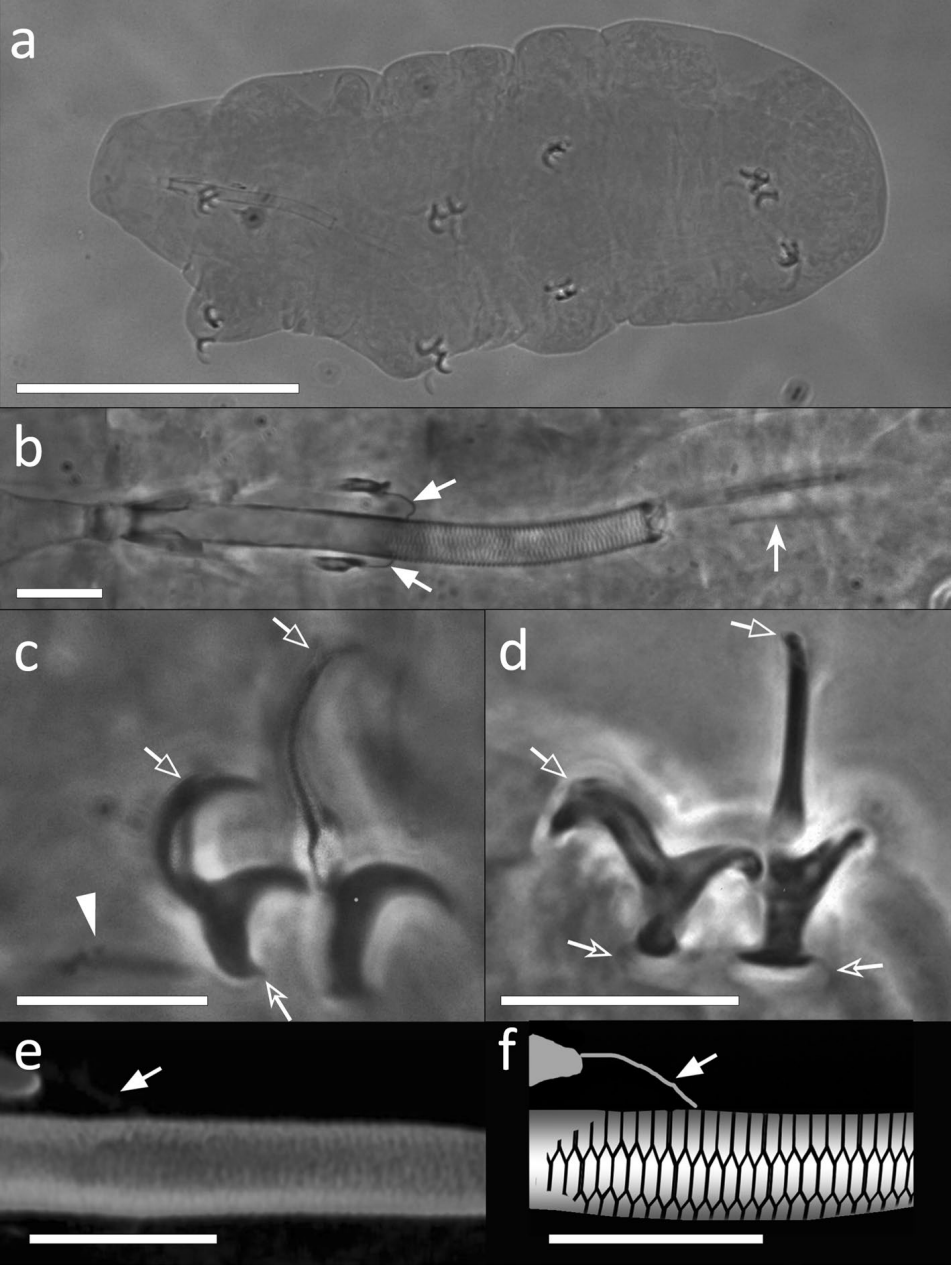
*Itaquascon magnussoni* sp. nov. (**a**) *In* toto (ventro-lateral view), (**b**) Bucco-pharyngeal apparatus (dorso-ventral view from multiplanar images stack), (**c**) Claw II (lateral view from multiplanar images stack), (**d**) Claw VI (frontal view), (**e**) First section of the pharyngeal tube (3D dorsal reconstruction), (**f**) First section of the pharyngeal tube (drawn of dorsal view). White arrows: stylet supports; indented arrow: thickened bar in the pharynx; empty arrows: accessory points of the main claw branch; empty indented arrows: pseudolunules; arrowhead: cuticular bar under the claw. (**a**–**c**) Holotype. (**a**–**d**) LM, PhC; (**e**) CLSM; (**f**) schematic drawing. Scale bars (**a**) 100 µm; (**b**–**d**) 10 µm, (**e**,**f**) 5 µm.

Body whitish, 135.9–509.3 µm in length (Fig. 5a). Eye-spots absent in mounted specimens. Cuticle smooth. Bucco-pharyngeal apparatus of *Itaquascon* type (Fig. 5b). Rigid and straight buccal tube, clearly longer than the apophyses for the insertion of the stylet muscles [AISM]. AISM symmetrical and flat ridge-shaped. Buccal tube followed by a pharyngeal tube almost of the same length (pharyngeal tube *pt* 91.6–113.8). Flexible pharyngeal tube formed by a rope-shaped thickening organized in a geometrical repeated pattern resembling an alternating hexagonal “wire meshes” (Fig. 5b,e,f); the “wire meshes” pattern begins in a more anterior position dorsally and ventrally than laterally (Fig. 5e,f). Very thin stylet supports present but hardly detectable and inserted on the pharyngeal tube in its anterior portion (Fig. 5b,e,f). Small stylet furca with short branches ending in drop-shaped condyles. Stylet coat more sclerotized in its proximal and distal portions than in its middle part. Pharyngeal tube ending within the pharynx with three small triangular apophyses (Fig. 5b). In the pharynx, only a single long, straight, and weakly thickened bar present (Fig. 5b).

Double-claws of *Hypsibius* type (Fig. 5c,d), internal (anterior, in the claw IV) and external (posterior, in the claw IV) claws of the same legs different both in shape and size. Claws increasing in length from the first to the fourth legs. Basal part of all claws long, with enlarged base. Main branch of external claw (posterior, in the claw IV) long, quite straight, and poorly sclerotized throughout its length, with evident accessory points; its proximal part placed on a cuticular digit and connected with the secondary branch with a pair of filaments spanning from the tip of the branch (Fig. 5c). Main branch of internal (anterior, in the claw IV) claw shorter and more curved than the external, with evident accessory points. Thin and hardly detectable pseudolunules present under all claws (Fig. 5c,d). Straight cuticular bars (Fig. 5c), similar in size, with ragged margins on the internal side of the legs I-III, extending from the internal claw base to the anterior side of the leg, weakly visible only on the first pair of legs.

Eggs unknown.

#### Differential diagnosis

*Itaquascon magnussoni* sp. nov. differs from all other *Itaquascon* species by having the stylet support inserted on the flexible pharyngeal tube. Considering the presence of the thickening within the pharynx, the most similar species of *I. magnussoni* sp. nov. are *Itaquascon placophorum* Maucci, 1973^47^ and *Itaquascon simplex* (Mihelčič, 1971)^48^ (considered *nomen dubium* by Ramazzotti et al.^49^, thus not considered in this diagnosis)*.*

*Itaquascon magnussoni* sp. nov. differs from *I. placophorum* by: the longer buccal tube with respect to the bucco-pharyngeal tube (buccal tube 16–17% of the bucco-pharyngeal tube in the holotype of *I. placophorum* and 48.0–48.4% in *I. magnussoni*), the longer thickening in the pharynx (calculated *pt* 31.3 in the holotype of *I. placophorum*; *pt* 44.8–63.5 in *I. magnussoni*), the claws with pseudolunules and evident accessory points on the main branch.

#### Molecular characterization

The analyses of the molecular markers were not possible due to the lack of alive specimens: the genomic material extracted from dead specimens gave no amplicons.

#### Etymology

The species name is to honor Sven-Erik Magnusson, a sustainability visionary and leading person behind the development of Kristianstads Vattenrike Biosphere Reserve, and the first Coordinator of the Biosphere Reserve.

**Thulinius gustavi sp. nov.**

ZooBank: lsid:zoobank.org:act:149B5B73-580F-4BBE-BCB0-BCFD53E54EB5.

#### Type locality

Araslövssjön Lake, Näsby Fält (Skåne, Sweden). Upper layer of freshwater sediments in the bottom of the shore of the lake (56.059050 N, 14.135425 E), sample SVC31 (C4343 in Bertolani’s Collection).

#### Type repositories

The holotype (SVC31 s3b) and nine paratypes (SVC31 s2, s4, s5; SVC31b s2, s6, s8) are at HKR, one paratype (SVC31 s4) is in the collection of the SMNH, and one paratype (C4343 s1) in the Bertolani’s Collection of the Unimore. Two paratypes were mounted on stubs for SEM observation.

#### Description

**Figure 6:**
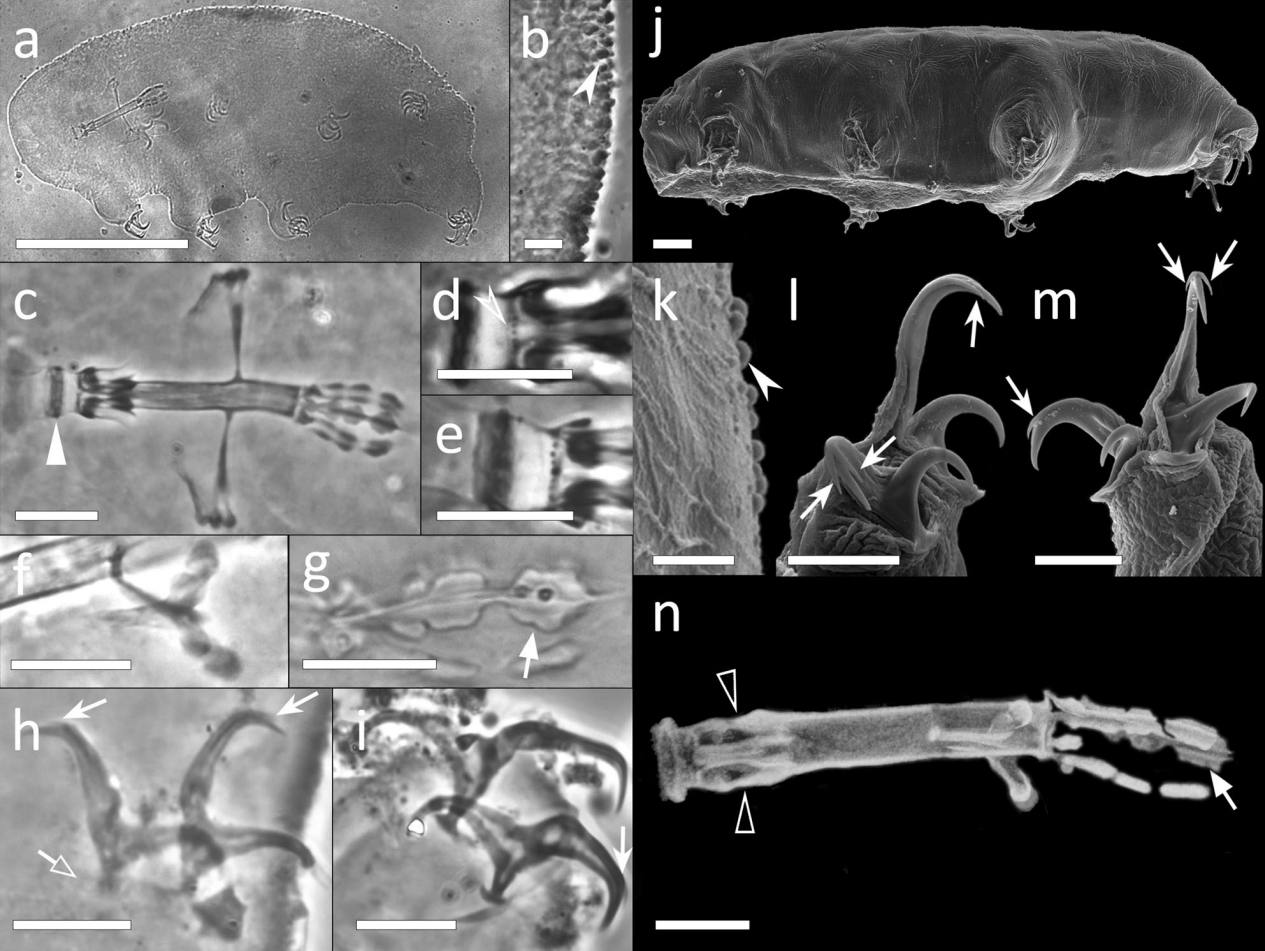
*Thulinius gustavi* sp. nov. (**a**) *In toto* (dorso-lateral view), (**b**) Cuticular ornamentation (dorso-lateral view), (**c**) Bucco-pharyngeal apparatus (dorso-ventral view from multiplanar images stack), (**d**) Buccal armature (dorsal view), (**e**) Buccal armature (ventral view), (**f**) Stylet furca (frontal view), (**g**) Macroplacoids (frontal view from multiplanar images stack), (**h**) Claw I (lateral view), (**i**) Claw IV (frontal view), (**j**) *In toto* (lateral view), (**k**) Cuticular ornamentation (dorso-lateral), (**l**) Claw II (frontal-lateral view), (**m**) Claw IV (frontal view), (**n**) Bucco-pharyngeal apparatus (3D lateral reconstruction). Indented arrowheads: cuticular ornamentation; white arrowhead: peribuccal lamellae; empty indented arrowhead: posterior line of small round teeth; white arrow: second macroplacoid constriction; white indented arrows: accessory points of the main claw branch; empty arrow: pseudolunules; empty arrowheads: apophyses for the insertion of the stylet muscles. (**a–c**) Holotype. (**a–f**), (**h,i**). LM, PhC; (**g**) LM, DIC; (**j–m**) SEM; (**n,o**) CLSM; Scale bars (**a**) 100 µm; (**b**–**j**,**n**). 10 µm; (**k**) 2 µm; (**l**,**m**) 5 µm.

Body whitish, 231.0–346.0 µm in length (Fig. 6a,j). Eye-spots present. Dorsal cuticle sculptured with large tubercles, with polygonal base, that gradually increase in size from the head to the posterior side of the body (Fig. 6b,k).

Bucco-pharyngeal apparatus with antero-ventral mouth opening (Fig. 6c,n). Buccal tube straight. Twelve peribuccal lamellae present. Buccal armature formed by a posterior line of small round teeth, followed by a line of large rounded teeth in the position of the transversal crests (Fig. 6d,e). Two bigger rounded teeth present ventrally within the second line in correspondence of the stylet sheaths (Fig. 6d). Ventral and dorsal AISM crest-shaped and symmetrical with respect to the frontal plane (Fig. 6n). Long and straight stylet supports with a distal flat enlargement. Typically-shaped stylet furca, with long branches provided with large apophyses (Fig. 6f). In the pharynx, large pharyngeal apophyses overlapping the first macroplacoid (Fig. 6n); three rod-shaped (in lateral view) macroplacoids arranged in a curved line; first and second almost fuse together, third spaced from the second (Fig. 6g). In frontal view, the first macroplacoid in shape of a triangle, second in shape of a rectangle with rounded corners, and the third almond-shaped and slightly constricted in the middle; length sequence 3 > 1 > 2.

Double-claws of *Isohypsibius* type (Fig. 6h,i,l,m) increasing in length from the first to the fourth pair of legs, external claw slightly longer than the internal. Basal portions of all claws short and slender, enlarged in their proximal portion. Primary branch of all claws with thin and short accessory points (never reaching the end of the branch and not always visible) and divided from the rest of the claw by a basal septum, with a dorsal knob-like thickening (Fig. 6h,i). Pseudolunules hardly detectable and small on external claws of legs I–III (Fig. 6h,l), larger on posterior claw of legs IV (Fig. 6i,m).

Smooth oval eggs laid in exuvium (an exuvium with four eggs was found).

#### Differential diagnosis

*Thulinius gustavi* sp. nov. differs from all other *Thulinius* species by having a dorso-lateral ornamented cuticle with tubercles. Considering the presence of pseudolunules only under the external (posterior, in claws IV) claws, the most similar species are *Thulinius romanoi* bertolani, bartels, Guidetti, Cesari & Nelson, 2014^50^ and *Thulinius saltarsus* (Schuster, Toftner & Grigarick, 1978)^51^.

*T. gustavi* sp. nov. differs from:

*T. romanoi* by: the absence of ornamented cuticle in ventral side, the presence of eye-spots, the more narrow buccal tube (*pt* 21.9 and 13.4 in the holotype and a paratype of *T. romanoi*; *pt* 9.1–10.7 in *T. gustavi*), the stylet support inserted more posteriorly (*pt* 62.9 in the holotype of *T. romanoi*; *pt* 70.2–73.1 in *T. gustavi*), the macroplacoid length sequence (1 > 3 > 2 in *T. romanoi*), the shorter accessory points that never reach the end of the branch, and the presence of evident pseudolunules in claw IV;

*T. saltarsus* by: the dorso-lateral ornamented cuticle, presence of eye-spots, the macroplacoid length sequence (1 > 3 > 2 in *T. saltarsus*).

#### Molecular characterization

The analyses of the molecular markers were not possible due to the lack of alive specimens: the genomic material extracted from dead specimens gave no amplicons.

#### Etymology

The species name has been chosen in honor of Gustav Thulin (1889–1945), the first internationally recognized Swedish tardigradologist, who made important contributions to the knowledge of the Swedish tardigrade fauna and to modern taxonomy and phylogenetics of tardigrades.

### Faunistic results

The analysis of the 34 samples collected (33 terrestrial and one freshwater; see “Methods”) in the five sampled areas within the KVBR revealed the presence of 33 morphospecies belonging to 20 genera (Table 3) of Eutardigrada (18 genera and 29 species of Parachela, and one genus and two species of Apochela), and Heterotardigrada (one genus, two species). The identification of the morphospecies was carried out with morphological and morphometric approaches.

**Table 3:** Morphospecies identified with a morphological approach in the samples collected from five areas within Kristianstads Vattenrike.

SVC #	# record	Balsberget							HKR campus					Sånnarna								
		1	2	3	4	7	8	9	10	11	12	13	14	15	16	17	18	19	20	21	22	23
Density (ind./g)		/	/	/	/	/	/	/	23.71	66.40	9.67	75.42	31.85	19.13	2.67	9.52	1.21	0.71	0.13	0.63	1.93	0.48
*Echiniscus spiniger* Richters, 1904^†^	1										0.33											
*Echiniscus testudo* (Doyére, 1840)^†^	5									15.20		4.58	13.33		0.22							
*Milnesium asiaticum* Tumarov, 2006^†^	11					X			1.43							0.48	0.13	0.08				
*Milnesium t. tardigradum* Doyere, 1840	6									2.80	2.00					0.24	0.04	0.08				
*Diphascon mitrense* Pilato et al., 1999 ‡	1																					
*Diphascon* sp*.*	1																				0.02	
*Hypsibius convergens* (Urbanowicz, 1925)	11	X	X	X		X										6.19				0.59	0.02	
*Hypsibius pallidus* Thulin, 1911	2		X												0.22							
*Adropion scoticum* (Murray, 1905)	2																				0.07	
*Itaquascon magnussoni* sp. nov.^‡^	3			X																		
*Mesocrista spitzbergensis* (Richters, 1903)	1																					
*Pilatobius o. oculatus* (Murray, 1906)^‡^	1					X																
*Pilatobius patanei* (Binda & Pilato, 1971)^‡^	2		X																			
*Ramazzottius oberhaeuseri* (Doyère, 1840)	13									8.00	5.00	70.83	18.52		2.44	2.38	0.63	0.08	0.13	0.04	0.29	
*Ramazzottius anomalus* (Ramazzotti, 1962)^‡^	3								6.57													
*Dianea sattleri* (Richters, 1902)	2			X													0.04					
*Isohypsibius marcellinoi* Binda & Pilato, 1971	1																					
*Ursulinius lunulatus* (Iharos, 1966)^†^	3			X			X															
*Thulinius gustavi* sp. nov.^‡^	1																					
*Macrobiotus lazzaroi* Maucci, 1986^‡^	3								15.14													
*Macobiotus macrocalix* Bertolani et al., 1993^†^	5					X																
*Macrobiotus persimilis* Binda & Pilato, 1972^‡^	10				X	X	X											0.39				
*Macrobiotus polonicus* Pilato et al., 2003^‡^	2									11.60												
*Macrobiotus* cf. *polonicus*	1									4.40												
*Macrobiotus trunovae* Biserov et al., 2011^‡^	6			X			X	X													0.31	
*Macobiotus* aff. *wandae*^‡^	4									5.20							0.04					
*Mesobiotus emiliae* sp. nov.^‡^	5		X				X														1.25	
*Minibiotus intermedius* (Plate, 1888)	5				X	X					0.33											
*Paramacrobiotus peteri* Pilato et al., 1989‡	1									0.80												
*Paramacrobiotus pius* Lisi et al., 2016^‡^	4	X					X										0.29					0.48
*Xerobiotus gretae* sp. nov.^‡^	2													19.13				0.08				
*Dactylobiotus dispar* (Murray, 1907)^†^	1																					
*Murryon dianeae* (Kristensen, 1982)^†^	1				X																	
Total morphospecies in each sample		2	4	5	3	6	5	1	3	7	4	2	2	1	3	4	6	5	1	2	6	1
Total morphospecies in each area		15							12					15								

SVC #	Gropahålet				Näsby Fält																	
	25	26	27	28	29	30	31	32	33	34	35											
Density (ind./g)	0.20	0.37	8.85	0.51	18.75	2.94	/	10.00	4.18	1.64	5.14											
*Echiniscus spiniger* Richters, 1904^†^																						
*Echiniscus testudo *(Doyére, 1840)^†^											0.39											
*Milnesium asiaticum *Tumarov, 2006^†^	0.07	0.28			0.42	0.20			1.39	0.25												
*Milnesium t. tardigradum *Doyere, 1840		0.02																				
*Diphascon mitrense *Pilato et al., 1999^‡^								0.50														
*Diphascon *sp*.*																						
*Hypsibius convergens *(Urbanowicz, 1925)	0.07	0.07					X		1.14													
*Hypsibius pallidus* Thulin, 1911																						
*Adropion scoticum* (Murray, 1905)			2.29																			
*Itaquascon magnussoni* sp. nov.^‡^			0.63					3.50														
*Mesocrista spitzbergensis* (Richters, 1903)			1.98																			
*Pilatobius o. oculatus *(Murray, 1906)^‡^																						
*Pilatobius patanei *(Binda & Pilato, 1971)^‡^							X															
*Ramazzottius oberhaeuseri *(Doyère, 1840)				0.04						1.15												
*Ramazzottius anomalus *(Ramazzotti, 1962)^‡^	0.07				0.21																	
*Dianea sattleri* (Richters, 1902)																						
*Isohypsibius marcellinoi *Binda & Pilato, 1971											0.17											
*Ursulinius lunulatus* (Iharos, 1966)^†^							X															
*Thulinius gustavi* sp. nov.^‡^							X															
*Macrobiotus lazzaroi *Maucci, 1986^‡^					0.21	0.98																
*Macobiotus macrocalix *Bertolani et al., 1993^†^					13.54	0.59		11.25			2.51											
*Macrobiotus persimilis *Binda & Pilato, 1972^‡^			2.29	0.31	1.25	0.98			1.65	0.16												
*Macrobiotus polonicus *Pilato et al., 2003^‡^											1.84											
*Macrobiotus *cf. *polonicus*																						
*Macrobiotus trunovae *Biserov et al., 2011^‡^			0.63							0.16												
*Macobiotus* aff.* wandae*^‡^								1.25		1.31												
*Mesobiotus emiliae *sp. nov.^‡^			0.10			0.20				0.25	0.06											
*Minibiotus intermedius *(Plate, 1888)				0.16				0.75														
*Paramacrobiotus peteri *Pilato et al., 1989‡																						
*Paramacrobiotus pius* Lisi et al., 2016^‡^																						
*Xerobiotus gretae *sp. nov.^‡^																						
*Dactylobiotus* *dispar *(Murray, 1907)^†^							X															
*Murryon dianeae *(Kristensen, 1982)^†^																						
Total morphospecies in each sample	3	3	6	3	5	5	5	5	3	6	5											
Total morphospecies in each area	12				19																	

The table provides name of the sampling areas, sample number, name of the identified morphospecies, number of samples in which morphospecies have been identified (# record), total density of tardigrades in each sample (ind/g dry substrate), density of each morphospecies within the sample (ind/g dry substrate), presence of the morphospecies (for Balsberget the density was not calculated), total number of morphospecies in each sample, and total number of morphospecies in each sampling area.

^†^New record for Skåne.

^‡^New record for Sweden.

^X^Morphospecies present.

The highest densities of tardigrades (ind/g) were found in a lichen and a moss (Table 3, Supplementary Table S4). However, tardigrade densities in both lichens (five samples) and mosses (13 samples) were highly variable, ranging from 2.7 to 75.4 ind/g (mean: 27.6 ind/g, SD: 29.5, N = 5) in lichens, and from 0.2 to 66.4 ind/g (mean: 11.1 ind/g, SD: 17.9, N = 13) in mosses. In contrast, leaf litter (two samples) and soil with grass (three samples) were less abundant in animals and had a more homogeneous density: 0.1–1.9 ind/g (mean: 0.9 ind/g, SD: 0.68, N = 5; Table 3, Supplementary Table S4).

The species belonging to the family Macrobiotidae were the most represented, found in 23 samples. The 11 macrobiotid species belonged to five genera (*Macrobiotus, Mesobiotus, Minibiotus*, *Paramacrobiotus,* and *Xerobiotus*) and were found in 69.7% of the terrestrial substrates, with variable diversity (1–4 species per substrate) and variable density (0.1–22.4 ind/g; Table 3) within each sample. The genus *Macrobiotus* was the most represented among the Macrobiotidae and among all the genera identified in all samples (7 species distributed among 17 samples).

The sample SVC11 (C4340 in Bertolani’s Collection) was the richer in terms of overall density (66.4 ind/g; Table 3). Within this sample *Macrobiotus polonicus* Pilato, Kaczmarek, Michalczyk & Lisi, 2003^52^ and *Macrobiotus wandae* Kayastha, Berdi, Mioduchowska, Gawlak, Łukasiewicz, Gołdyn, & Kaczmarek, 2020^53^ were initially morphologically identified, but the evidence of intraspecific variability for some characters led us to suspect the presence of cryptic species. The analyses were performed by genotyping the markers ITS2 and *cox1*. The analyses of the *cox1* were unsuccessful, but the ITS2 sequences amplified from nine specimens (C4340 C–D, J–P; GenBank accession numbers: XXXX) were sufficient to reveal the presence of three species: *Macrobiotus polonicus, Macrobiotus* cf. *polonicus*, and *Macrobiotus* aff. *wandae*. *Macrobiotus polonicus,* already identified via morphology, was confirmed also by a very low p-distance of its sequences (0.00–0.01%; 587 bp) with respect to those already attributed to this species (Supplementary Table S3). In the population, eight males with spermatozoans within the gonad were found. One specimen previously identified as *M. polonicus* was revealed to belong to a cryptic taxon that we named *M*. cf. *polonicus* (p-distance 0.04–0.05% with respect to *M. polonicus* sequences; Supplementary Table S3). *Macrobiotus* cf. *polonicus* differs morphologically from *M. polonicus* by the presence of fine granules on the external side of all legs, for this species the egg morphology is unknown. *Macrobiotus* aff. *wandae* is probably a species new to science both for the ITS2 differences (p-distance 0.17–0.18% from the three available sequences of *Macrobiotus wandae*; Supplementary Table S3) and for the different shape of the egg having a more expanded distal disk on the processes. Since only one egg and few animals of this species were collected and the *cox1* sequencing gave no result, further collection and analyses will needed before a possible new species description.

The most common morphospecies in the samples was *Ramazzottius oberhaeuseri* (Doyére, 1840)^54^. It was retrieved from 39.4% (13 terrestrial samples) of the samples and from all the sampled areas except Balsberget, with a highly variable density: e.g., 70.8 ind/g in a lichen, 0.3 ind/g in a soil with grass, and 0.1 ind/g in a moss or in a leaf litter. *Milnesium asiaticum* Tumanov, 2006^55^ was found in 33.3% (11 samples) of the terrestrial samples from all the sampled areas, but with low density (0.1–1.4 ind/g). *Hypsibius convergens* (Urbanowicz, 1925)^56^ was found in 32.4% (11 samples) of both terrestrial and freshwater samples, and in all the sampled areas except the HKR campus, with a low density (0.1–6.2 ind/g). *Macrobiotus persimilis* Binda & Pilato, 1972^57^ had a wide distribution, found in 30.3% (10 samples) of terrestrial samples, with low density (0.3–2.3 ind/g). All the other morphospecies have a more restricted distribution within the samples (Table 3).

Considering all samples, the species diversity within individual samples and between sampling areas was variable (number of morphospecies: 0–7; 12–19, respectively), but most of the samples (67.6%) had three to six morphospecies, and only within two samples (SVC5, 6; 0.5%) there were no tardigrades (Table 3).

## Discussion

The species abundance of tardigrades has been shown to be mostly related to the sampling effort, rather than to the surface of the sampling area^58^. The limited number of visited sites and limited sampling effort (34 samples collected in five sites of a 1050 km^2^ area) of our study suggest that the true tardigrade biodiversity of KVRB is much higher than recorded, and that there are considerably more tardigrade species to be found in this area. Moreover, the identifications of species were carried out mainly morphologically and hidden molecular diversity, linked to cryptic species, may have been undervalued. Nonetheless, this study documented 33 tardigrade species from the KVBR (Table 3), of which 22 are new records for Skåne, 15 are new records for Sweden, and four are new to science.

KVRB has a higher biodiversity of tardigrades (33 species in 34 samples; n° species / n° samples ratio 0.97) compared to areas similar in types of analysed substrates and sampling effort. As comparison, thirty-nine species (found in 64 samples; ratio 0.61) that were collected in a much smaller area (Monte Rondinaio Valley, Apennines, Northern Italy)^59^ which was considered an area of high diversity^60^. Other faunistic studies, comparable in sampling effort and substrate diversity, found lower diversity, e.g. 26 species in 32 samples, ratio 0.81 (Marche-Umbria, Italy)^61^, 23 species in 48 samples, ratio 0.47 (Sardinia, Italy)^62^, 41 species in 60 samples, ratio 0.68 (Great Smoky Mountains National Park, TN-NC, USA)^60^, 10 species in 13 samples, ratio 0.76 (Jyväskylä, Finland)^58^. Therefore, this study indicates a high biodiversity of tardigrades in the KVBR, as suggested by the presence of many genera, species, and new taxa from different environments and habitats (i.e. freshwater sediments, soil, and moss). Additional sampling efforts in the KVBR area will likely reveal many more species. For example, in Bartels & Nelson^13^ an increase of about six times in the sampling effort in the same sampling area (Great Smoky Mountains National Park, USA)^60^ led to the identification of 78% more species, to a final total amount of 73 species in 401 samples, with a decrease in ratio to 0.18.

The description of *X. gretae* sp. nov. and *I. magnussoni* sp. nov. increases the number of species in *Xerobiotus* and *Itaquascon* and therefore enriches the knowledge of the synapomorphic characters of these phylogenetic lines. *Xerobiotus gretae* sp. nov. presents an area of the legs that surrounds the claws, corresponding to what we called “garter-like structure”, that is present also in *X. xerophilus*. This structure was described for *X*. *xerophilus* by Dastych and Alberti^42^ as “small swelling” (see SEM Figs. 8, 12, 17 in Dastych and Alberti^42^) and is visible in the LM Fig. 6e in Pilato et al.^43^. Furthermore, in the original description of *X. pseudohufelandi* by Iharos^44^ the garter-like structures are not reported, but they are visible in Fig. 6b in Pilato et al.^43^. Similarly, in *X. euxinus* this structure is not reported in the original description, but a pale refractive structure is visible in the leg shown in Fig. 5b in Pilato et al.^43^. Additionally, this garter-like structure, the smaller legs of the first pair compared to those of the second and third pairs, and the absence of ventral cuticular pores were always observed in three *Xerobiotus* populations from Portugal, France, and Italy (Guidetti R. personal observation). Smaller legs of the first pairs are also present in *X. xerophilus* (see Figs. 1, 6–8 in Dastych and Alberti^42^).

Recently, the genus *Xerobiotus* has been proposed to be suppressed based on molecular analyses that would nest this genus within the *Macrobiotus* species^45^. In our opinion, the *Xerobiotus* suppression is premature (or not sufficiently supported) based on the presence of several clear synapomorphies of *Xerobiotus* species (see below) that allow distinguishing the genus from any other tardigrade genera. *Macrobiotus* (according to Stec et al.^45^), the clade including *Xerobiotus*, is still not morphologically well defined and inhomogeneous (e.g., in claw and egg morphologies), is without any autapomorphy, and contains several lineages (one of which is composed by *Xerobiotus* species) recognized by molecular data, but to date not yet supported by morphological apomorphies^45^ (excluding *Xerobiotus*). Moreover, the suppression of *Xerobiotus* would also imply the suppression of the genus *Pseudohexapodibius* that shares with *Xerobiotus* the morphology of claws of the first three pairs of legs and of the bucco-pharyngeal apparatus, together with the reduction of the leg size. Such important changes in the systematics (e.g., the erection and suppression of genera) need well supported results from both morphological and molecular point of view. According to our new findings, the garter-like structures are an additional apomorphy of *Xerobiotus*. This genus hence differs from the other genera of Macrobiotoidea in several synapomorphic characters, such as the presence of: smaller legs of the first pairs compared to those of the second and third pairs, garter-like structures (i.e. swelling of the leg with microdigitations) in each leg, different shape of the claws between the hind legs and those of the first three pair of legs, typical shape of the claw (of *Xerobiotus* type and without lunules) in the first three pair of legs, claws of the hind legs with long common tact and short branches, and very small cuticular pores only in the dorsolateral portion of the cuticle. Stec et al.^45^, in the new definition of *Macrobiotus*, specified that the former *Xerobiotus* species have special unique characters that differentiate *Xerobiotus* from all the other *Macrobiotus* species. Thus, according to Stec et al.^45^, all the *Macrobiotus* species share the same claw type apart from the species of the former genus *Xerobiotus*, further underlining the uniqueness of the characters of these species. Several new genera have been erected from *Macrobiotus*, since it was the first genus created in the phylum, see^1^, and probably other genera will be identified with related apomorphies, explaining the presence of several phylogenetic lineages in the cluster identified by Stec et al.^45^ as *Macrobiotus.*

*Itaquascon* (Hypsibiidae) is a genus belonging to the subfamily Itaquasconinae, i.e. eutardigrades with *Hypsibius* type of claws, a bucco-pharyngeal apparatus subdivided into a rigid buccal tube and a flexible pharyngeal tube provided with or without spiral thickening, and AISM in shape of wide flat ridges^46,63,64^. The thickenings in the pharyngeal tube do not always form a true annulation in the Itaquasconinae, but it is more likely a patterned thickening (i.e. simple, complex, net-like)^64^. According to Gąsiorek et al.^64^, the *Itaquascon* pharyngeal thickening pattern is formed by annuli forking and merging irregularly and vanishing in the ventral side (net-like). In contrast with their observation, the net-like thickening pattern of *I. magnussoni* sp. nov. and *I. placophorum* (in type material and other populations; see “Methods”) that we observed resembles an alternating hexagonal wire mesh (Fig. 6e,f). Thus, our observations suggest that the pattern of the pharyngeal tube thickenings has a major variability within the genus in contrast to that pointed out by Gąsiorek et al.^64^. Analyses of more species, observing also type material, are needed to better understand the ultrastructure of the flexible pharyngeal tube of each genus of this subfamily. In addition, in the amended diagnosis of *Itaquascon* by Gąsiorek et al.^64^, it is reported that this genus is “devoid of placoids”, but in *I. placophorum* (and *I. magnussoni* sp. nov.) thickenings (a.k.a. bulbar linings, cuticular ridges) within the pharynx are present (Supplementary Figure S1). For this reason, we propose an amended diagnosis for the genus *Itaquascon* (see “Taxonomic account”)*.*

Moreover, the observation of the holotype of *Platicrista itaquasconoide* (Durante Pasa & Maucci, 1975)^21^ (slide: CT3552) revealed the presence of a lateral patterned thickening anteriorly to the stylet support insertion point (Supplementary Figure S1). This newly observed character and all the other characteristics of the species fit with the recently described genus *Meplitumen* Lisi et al.^65^. Thus, we propose to move the species, previously moved from *Diphascon* to *Platicrista*^66^, to this genus with the new name *Meplitumen itaquasconoide* comb. nov.

The presence of 29 morphospecies and the four new species identified in this study increased the known tardigrade biodiversity in Sweden by 11%. Furthermore, four new type localities are added to the 21 previously reported in literature for Sweden^24^. Thus, four of the 25 Swedish type localities (16%) and 33 of the 116 recorded Swedish species (28%) are located within the KVBR area. Despite the restricted sampling effort of this study, our study suggests that the KVBR conceals a very high diversity of tardigrades. In particular, further studies of the sandy habitats and the diverse freshwater environments in the KVBR area will likely reveal additional species, some of which may also be new to science. The new species found in this study increased our knowledge of global tardigrade diversity (more than 1330 species are now described^1^), allowed the identification of new synapomorphic characters for a better definition of the taxa, and illustrated that a broad faunistic survey also can have an impact on the systematics of a phylum.

### Taxonomic account

#### Itaquascon de Barros, 1939 (amended diagnosis)

Cuticular thickening between the buccal tube and the pharyngeal tube absent in the known species. Sinusoidal stylet supports. Pharynx elongated. Cuticular thickenings within the pharynx present or absent. Claws of *Hypsibius* type, with external claws with primary branches markedly longer than secondary ones.

## Methods

### Tardigrade sampling and observations

Thirty-four samples of mosses, leaf litter, soil with grass, and one freshwater detritus sample from five different sampling areas within the KVBR (i.e. Balsberget, Gropahålet, Kristianstad University Campus, Näsby Fält, Sånnarna) were collected in June 2014 (Supplementary Table S4). Extraction of tardigrade animals and eggs were carried out using sieves (mesh size: 250 μm and 38 μm) after keeping the samples in distilled water for about 30 min. Specimens were isolated from the extraction using a needle and a Pasteur pipette under a stereo microscope, and then mounted on slides in Hoyer’s or Faure-Berlese fluid for observations with LM and Confocal Scanning Laser Microscopy [CLSM]. Additional specimens were observed with SEM following the protocol of Guidetti et al.^7^.

Dry weight of mosses, lichens, soil with grass, and leaf litters were estimated after the tardigrades extraction by drying the samples in an oven at 50 °C for four days, and the density of tardigrades per sample was calculated as the number of tardigrades per gram of dry substrate.

Remaining subsamples were stored dry at room temperature at the Department of Environmental Science and Bioscience at HKR or/and at the Department of Life Science of Unimore.

Five specimens of *Xerobiotus gretae* sp. nov. were recovered in 2019 for SEM observation from a permanent slide mounted with Faure-Berlese fluid in 2014 with the following new protocol: in order to recover the specimens within the mounting medium, the slide was rinsed in distilled water overnight to rehydrate and dilute the Faure-Berlese fluid. The coverslip was then gently removed using a needle and the specimens were recovered with a glass pipette. The recovered specimens were washed in distilled water three times. Subsequently, the protocol of Guidetti et al.^7^ was applied for preparation of specimens for SEM.

Observations with SEM (Nova Nano SEM 450, FEI company) and CLSM (Ti2-e with A1R HD Resonant scanning module, Nikon) were performed at the “Centro Interdipartimentale Grandi Strumenti” at Unimore. For the observation and 3D reconstruction with CLSM, the protocol of Guidetti et al.^7^ was used. Observations with LM and measurements were carried out under both phase contrast [PhC] and differential interference contrast [DIC] up to the maximum magnification (100 × oil objective) with an Olympus BX60 microscope equipped with an INFINITY 1 (Lumenera Corp.) digital camera and the image analysis software INFINITY ANALYZE 6.0 (Lumenera Corp.), at the Department of Environmental Science and Bioscience, Kristianstad University, and a Leica DM RB microscope equipped with AmScope MU1803 digital camera and the image analysis software AmScope v.4.11 (AmScope), at Unimore.

The body length of the animals was measured excluding the hind legs; the buccal tube length was measured from the anterior end (at the level of the stylet sheath, or where generally the transversal crests are present) to its posterior end within the pharynx; the claws were measured only if they were in perfect frontal view. Claws of *Itaquascon* specimens were measured following Pilato et al.^67^, those of *Mesobiotus* and *Xerobiotus* following Kaczmarek et al.^35^, those of *Thulinius* following Beasley et al.^68^. The common tract of the posterior claw of the legs IV of *Xerobiotus* was measured from the base of the claw, excluding lunule, to the branching point of the secondary branch. Raw morphometric data are given in the Supplementary Table S1 and are organized and analysed using the template Parachela ver.1.6 by Michalczyk et al.^69^ modified to perform the correction of the body size effect with the Thorpe’s normalization of the measures. Thorpe’s normalization calculation was carried out following the protocol from Bartels et al.^70^.

To confirm the identification and for further morphological investigations, type material (underlined) and non-type material of the Maucci’s Collection (at the Natural History Museum of Verona, Italy) were observed with LM and/or CLSM: *P. itaquasconoide* (CT3552); *I. placophorum* (CT1427–CT1430, CT12769); *X. pseudohufelandi* (CT8388–CT8391–CT8392–CT8396).

### Genotyping

The genotypization was performed on selected specimens from the samples SVC11, 15, 22, 31, 32 (Supplementary Table S5) for both the molecular characterization of the species new to science and for the detection of cryptic species. Before molecular analysis, animals and eggs were observed individually with LM following the protocol described by Guidetti et al.^7^ allowing us to obtain pictures of the specimens (photographic voucher specimens).

In particular, the evaluation of the presence of cryptic species was performed on the specimens belonging to the sample SVC11. In order to ensure the connection between the morphology of specimens and the DNA sequences, eggs were isolated and the newborns were left to hatch. Then, eggshells were mounted on permanent slides with Faure-Berlese fluid (hologenophore) and pictures of the hatched newborns were taken before the DNA extraction^28^.

Genomic DNA was extracted with QuickExtract kit (Epicentre) following the manufacturer protocol on live and dead specimens. Afterwards, when possible, the carcasses of the animals were recovered and placed on permanent slides (voucher specimens; available at the Department of Life Science of Unimore). For DNA extraction, four additional specimens of *Xerobiotus gretae* sp. nov. were recovered from the permanent slides C4341s1 following the protocol of Guidetti et al.^7^.

Molecular investigations were carried out using fragments of the nuclear ITS2, 18S, and 28S genes and the mitochondrial cytochrome oxidase 1 (*cox1*) gene (Supplementary Table S5). Several couples of primers and amplification protocols^2,46,71,72^ were tested (Supplementary Table S5). The amplified products were gel purified using the Wizard Gel and PCR cleaning kit (Promega, Madison, WI, USA). Sequencing reactions were performed using the ABI Prism Big Dye Terminator v. 1.1 sequencing kit (Applied Biosystems, Foster City, CA, USA) on purified amplicons. Each sequencing reaction contained 0.2 mM of a single PCR primer to initiate the sequencing reaction, 2 µl of BigDye, 70 ng of purified products, 4 µl of sequencing suffer (BigDye Terminator v. 1.1, Applied Biosystems, Foster City, CA, USA), and distilled water for a final volume of 20 µl. Cycling conditions for sequencing reactions consisted of 25 cycles of 96 °C for 10 s, 50 °C for 5 s, and 60 °C for 4 min. Both strands were sequenced using an ABI Prism 3100 (Applied Biosystems, Foster City, CA, USA). Nucleotide sequences of the newly analysed specimens were submitted to GenBank, and accession numbers are reported in the Supplementary Table S5. Chromatograms obtained and nucleotide sequences were checked by visual inspection and the sequences were aligned with the MAFFT algorithm. In order to perform proper molecular comparisons, we included sequences from GenBank pertaining to other specimens in our analysis. Pairwise nucleotide sequence divergences between scored haplotypes were calculated using p-distance by using MEGA7^73^.

## Supplementary Information

Supplementary Information 1.

Supplementary Information 2.

Supplementary Information 3.

Supplementary Information 4.

Supplementary Information 5.

Supplementary Information 6.

## Data Availability

All data generated and analysed during this study are included in this published article (and its Supplementary Information files). DNA sequences are deposited and available on GenBank.
