# Supplementary figures and images for: Tardigrades of Kristianstads Vattenrike Biosphere Reserve with description of four new species from Sweden
Source: Sci Rep. 2021 Mar 1;11:4861. doi: 10.1038/s41598-021-83627-w (PMC7921132; doi:10.1038/s41598-021-83627-w)

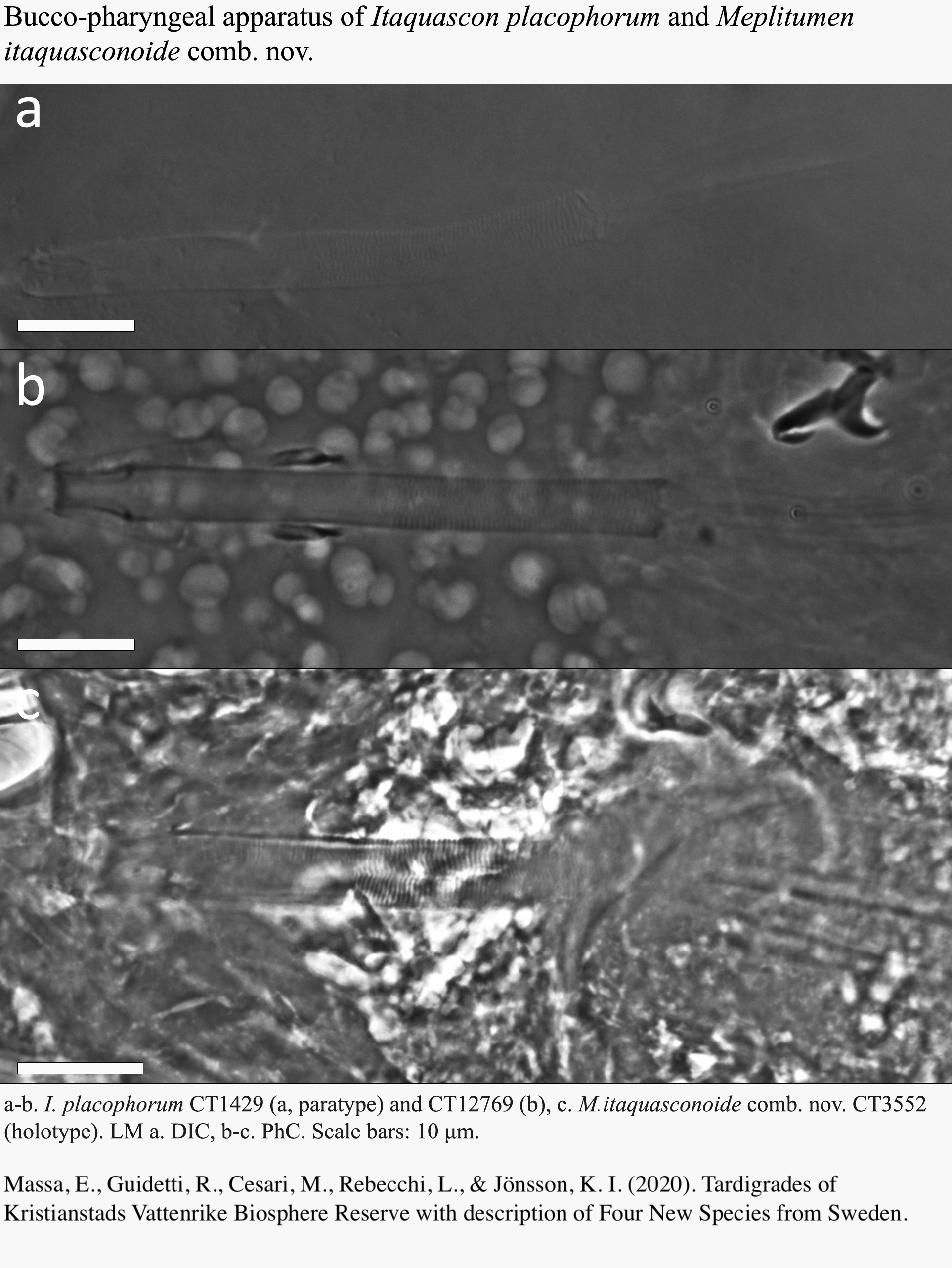

Supplement: Supplementary file 6 — Supplementary Information 6. [file 41598_2021_83627_MOESM6_ESM.tif]
